# A Nanostructured Lipid System as a Strategy to Improve the *in Vitro* Antibacterial Activity of Copper(II) Complexes

**DOI:** 10.3390/molecules201219822

**Published:** 2015-12-16

**Authors:** Patricia B. da Silva, Bruna V. Bonifácio, Regina C. G. Frem, Adelino V. Godoy Netto, Antonio E. Mauro, Ana M. da Costa Ferreira, Erica de O. Lopes, Maria S. G. Raddi, Tais M. Bauab, Fernando R. Pavan, Marlus Chorilli

**Affiliations:** 1 School of Pharmaceutical Sciences, UNESP—University Estadual Paulista, Campus Araraquara, Araraquara, 14801-902 São Paulo, Brazil; patrbent@yahoo.com.br (P.B.S.); brunavidalb@gmail.com (B.V.B.); ericalopes276@gmail.com (E.O.L.); raddims@fcfar.unesp.br (M.S.G.R.); tmbauab@gmail.com (T.M.B.); 2 Chemistry Institute, UNESP—University Estadual Paulista, Campus Araraquara, Araraquara, 14800-060 São Paulo, Brazil; rcgfrem@gmail.com (R.C.G.F.); adelino@iq.unesp.br (A.V.G.N.); mauro@iq.unesp.br (A.E.M.); 3 Chemistry Institute, USP—University São Paulo, Campus São Paulo, São Paulo, 05508-900 São Paulo, Brazil; amdcferr@iq.usp.br

**Keywords:** copper(II) complexes, antibacterial activity, *Staphylococcus aureus*, *Escherichia coli*, nanostructured lipid system

## Abstract

The aim of this study was to construct a nanostructured lipid system as a strategy to improve the *in vitro* antibacterial activity of copper(II) complexes. New compounds with the general formulae [CuX_2_(INH)_2_]·nH_2_O (X = Cl^−^ and *n* = 1 (**1**); X = NCS^−^ and *n* = 5 (**2**); X = NCO^−^ and *n* = 4 (**3**); INH = isoniazid, a drug widely used to treat tuberculosis) derived from the reaction between the copper(II) chloride and isoniazid in the presence or absence of pseudohalide ions (NCS^−^ or NCO^−^) were synthesized and characterized by infrared spectrometry, electronic absorption spectroscopy, electron paramagnetic resonance (EPR) spectroscopy, elemental analysis, melting points and complexometry with 2,2′,2′′,2′′′-(Ethane-1,2-diyldinitrilo)tetraacetic acid (EDTA). The characterization techniques allowed us to confirm the formation of the copper(II) complexes. The Cu(II) complexes were loaded into microemulsion (MEs) composed of 10% phase oil (cholesterol), 10% surfactant [soy oleate and Brij^®^ 58 (1:2)] and 80% aqueous phase (phosphate buffer pH = 7.4) prepared by sonication. The Cu(II) complex-loaded MEs displayed sizes ranging from 158.0 ± 1.060 to 212.6 ± 1.539 nm, whereas the polydispersity index (PDI) ranged from 0.218 ± 0.007 to 0.284 ± 0.034. The antibacterial activity of the free compounds and those that were loaded into the MEs against *Staphylococcus aureus* ATCC^®^ 25923 and *Escherichia coli* ATCC^®^ 25922, as evaluated by a microdilution technique, and the cytotoxicity index (IC_50_) against the Vero cell line (ATCC^®^ CCL-81^TM^) were used to calculate the selectivity index (SI). Among the free compounds, only compound **2** (MIC 500 μg/mL) showed activity for *S. aureus*. After loading the compounds into the MEs, the antibacterial activity of compounds **1**, **2** and **3** was significantly increased against *E. coli* (MIC’s 125, 125 and 500 μg/mL, respectively) and *S. aureus* (MICs 250, 500 and 125 μg/mL, respectively). The loaded compounds were less toxic against the Vero cell line, especially compound **1** (IC_50_ from 109.5 to 319.3 μg/mL). The compound **2-** and **3**-loaded MEs displayed the best SI for *E. coli* and *S. aureus*, respectively. These results indicated that the Cu(II) complex-loaded MEs were considerably more selective than the free compounds, in some cases, up to 40 times higher.

## 1. Introduction

The ability of bacteria to acquire resistance to drugs that are used as therapeutic agents has become a significant problem because there is a daily increase in the microbial resistance to the currently used antibiotics [1]. The prospects for the use of antimicrobial drugs in the future are still uncertain and require the search for new compounds with potential activity against pathogenic bacteria and fungi [2].

Copper is the third most abundant transition element in the human body and is present in the active site of enzymes that participate in oxidative reactions, such as cytochrome oxidase and superoxide dismutase. Furthermore, it has long been recognized that copper compounds are capable of inhibiting a wide variety of fungi and bacteria [3,4].

The Cu(II) ion has a tendency to interact with molecules to yield coordination compounds with different properties, including antibacterial activity. A coordination compound or metallic complex is the product of a Lewis acid-base reaction in which neutral molecules or anions (called ligands) bind to a central metal atom (or ion) by coordinate covalent bonds [5].

There has been an increasing interest in the literature on the investigation of copper(II) complexes with antimicrobial activity [6]. The compound [Cu(TTA)_2_], TTA = 2-tenoyltrifluoroacetone, which was synthesized and characterized by Xu *et al.* [7], showed antibacterial activity against *Escherichia coli* and *Staphylococcus aureus* with MICs (minimum inhibitory concentrations) of 180 and 150 μg/mL, respectively. Pahontu *et al.* [8] studied the antibacterial activity of the copper(II) compound [Cu(L)(ClO_4_)(H_2_O)] (HL = 4-[(*E*)-(2-hydroxy-4-methoxyphenyl)methyleneamino]benzoate) against *E. coli* and *S. aureus* and showed MICs of 500 μg/mL for both bacteria.

The low solubility of metallic compounds in water makes the majority of biological activity assays impossible, particularly *in vivo* experiments. Therefore, researchers have sought alternatives to produce these metallic compounds through technological strategies to effectively compartmentalize this diverse group of molecules and modify their behavior in the body [9]. Innovations in the field of drug delivery systems have emerged at a much faster pace over the past two decades. The improvements in therapeutic adherence and the efficacy of the treatment are important benefits in the search for innovative pharmacological formulations [5]. The microemulsion (ME) drug delivery system has been very important in the pharmaceutical field as it can provide a modern, more efficient therapeutic alternative that can be used for substances that are normally of limited use due to their hydrophobicity, toxicity or inability to access the site of action [10,11,12].

**Figure 1:**
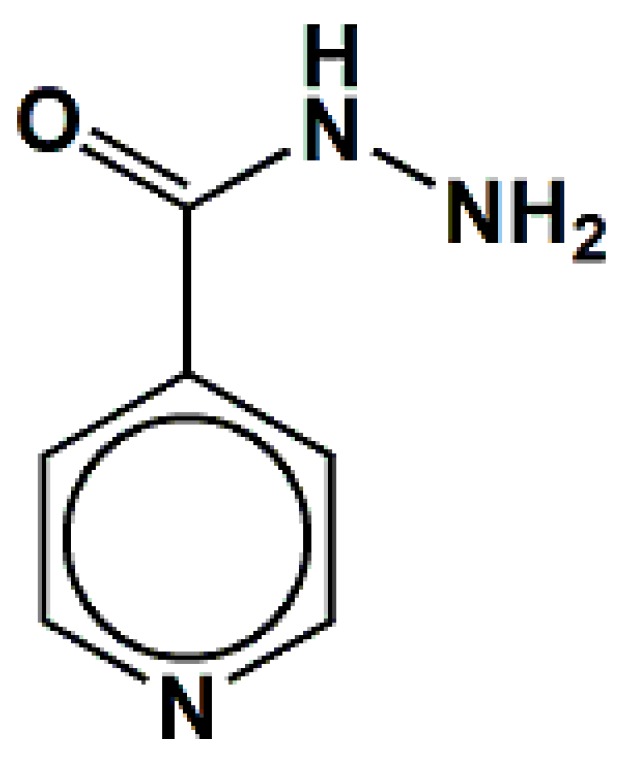
Structure for the isoniazid (INH).

**Figure 2:**
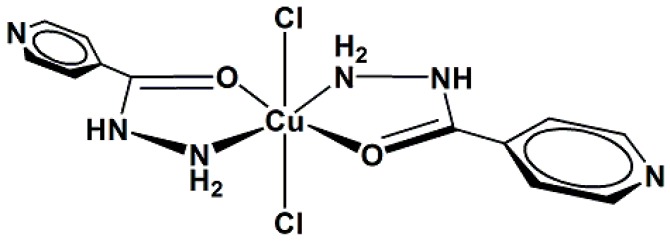
Proposed structure for the [CuCl_2_(INH)_2_]·H_2_O complex (**1**).

**Figure 3:**
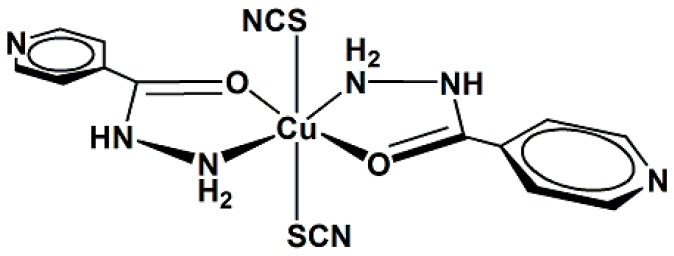
Proposed structure for the [Cu(NCS)_2_(INH)_2_]·5H_2_O complex (**2**).

**Figure 4:**
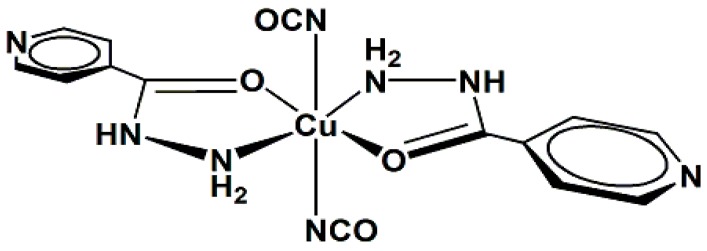
Proposed structure for the [Cu(NCO)_2_(INH)_2_]·4H_2_O complex (**3**).

This study reports the preparation and spectroscopic characterization of three copper(II) complexes containing isoniazid (INH) as a ligand, Figure 1: [CuCl_2_(INH)_2_]·H_2_O (**1**), Figure 2, [Cu(NCS)_2_(INH)_2_]·5H_2_O (**2**), Figure 3 and [Cu(NCO)_2_(INH)_2_]·4H_2_O (**3**), Figure 4, and the incorporation of these compounds into a nanostructured lipid system consisting of 10% phase oil (cholesterol), 10% surfactant [soy oleate and Brij^®^ 58 (1:2)] and 80% aqueous phase (phosphate buffer pH = 7.4). Then, the antimicrobial activity of the Cu(II) compounds was evaluated on two different bacterial strains (*Staphylococcus aureus* and *Escherichia coli*) and tested on VERO cells (a normal eukaryotic cell) to determine their cytotoxicity (IC_50_).

## 2. Results and Discussion

### 2.1. Chemistry

#### 2.1.1. Infrared (IR) Spectroscopy Studies

IR spectroscopy is one of the most widely used physical methods for inferring the binding mode of the isoniazid ligand. The most important IR frequencies of the new copper(II) complexes and their assignments are presented in Table 1.

The isoniazid (INH) molecule can be coordinated by the nitrogen atoms of the -NH_2_ group and/or pyridine ring as well as by the carbonyl oxygen. IR spectroscopy allows us to obtain valuable information regarding the participation of these groups in the coordination. When the nitrogen of the -NH_2_ group is involved in the coordination, there is a negative shift in the νNH mode (Δ = ν_ligand_ − ν_complex_) [13]. The coordination by the oxygen of the carbonyl group is suggested by the positive shift in the ν(C = O) mode (Δ = ν_ligand_ − ν_complex_) [14]. Some of the observed vibrations of the pyridine ring in the range of 1600–1400 cm^−1^ exhibit small shifts to higher frequencies after the formation of the complex [15].

**Table 1:** Selected vibrational data (cm^−1^) and assignments for isoniazid (INH) and the Cu(II) compounds (**1**–**3**).

Compound				Assignment
INH	1	2	3	
-	3437 *m*	3434 *m*	3449 *w*	νOH
3306 *m*, 3213 *sh*	3222 *s*	3241 *s*	3236 *w*	ν_as_NH_2_
3112 *s*	3098 *s*	3101 *sh*	3108 *w*	ν_si_NH_2_
3035 *m*, 3014 *m*	3048 *s*, 3019 *m*	3057 *sh*	3061 *sh*, 3017 *w*	νCH
-	-	-	2212 *s*	ν_as_NCO
-	-	2130 *s*	-	ν_as_NCS
1667 *s*	1653 *m*	1606 *w*	1615 *m*	νC=O
1635 *m*	1648 *s*	1648 *m*	1655 *sh*	*Scissoring* NH_2_
1603 *m*	1590 *s*	1603 *s*	1589 *sh*	ν_ring_ + δCH +δHOH
1556 *s*	1547 *s*	1554 *m*	1537 *m*	ν_ring_
1492 *w*	1494 *w*	1498 *w*	1499 *w*	δCH + ν_ring_ + δNH
1412 *m*	1415 *m*	1415 *m*	1416 *m*	δCH + ν_ring_
-	-	-	1317 *w*	ν_s_NCO
1335 *s*	1332 *m*	-	-	*Rocking* NH_2_
-	1284 *w*	1268 *w*	1283 *w*	ν_ring_
1221 *m*	1225 *m*, 1202 *s*	1233 *w*, 1208 *w*	1239 *w*	δCH + ν_ring_
1141 *m*	1139 *m*	1131 *w*, 1098 *w*	1131 *w*, 1103 *w*	δCH + ν_ring_ + νCN
1060 *s*	1065 *w*	1058 *m*	1058 *w*	ν_ring_ + δCH + δ_ring_
996 *m*	996 *w*	988 *w*	987 *sh*	ν_ring_ + δ_ring_
943 *s*	-	941 *w*, 919 *w*	945 *w*, 924 *w*	νNN + *twisting* NH_2_ + νNH_2_
888 *w*, 845 *m*	906 *w*, 881 *w*, 858 *m*	876 *w*	868 *sh*	γCH + γC-CO-NH-NH_2_
747 *w*	761 *w*	755 *w*	775 *w*, 756 *w*	γCH + τ_ring_ + γCO +ν NC-S
-	712 *m*	715 *m*	719 *w*	τ_ring_ + γCO + γCH
675 *s*	-	690 *m*	692 *w*	δ_ring_ + νC-CO-NH-NH_2_
659 *s*	-	664 *sh*	658 *w*	δ_ring_ + δCH
-	-	-	620 *w*	δNCO
504 *w*	538 *w*	504 *w*	501 *sh*	τHNNH + γNH + δNCC + δC-CO-NH-NH_2_ + δOCC
436 *w*	463 *w*	466 *w*, 430 *w*	420 *w*	γNH + τHNNH + γCH + τ_ring_ + δNCS

ν = stretching; δ = in-plane bending; γ = out-of-plane bending; *as* = asymmetric; *si* =symmetric; *s* = strong, *m* = medium, *w* = weak, *sh* = shoulder.

The IR spectra of complexes **1**–**3** revealed that the νNH band showed a shift of 3213 cm^−1^ (ligand free) to 3222 ($\Delta \bar{v}$ = −9 cm^−1^), 3241 ($\Delta \bar{v}$ = −28 cm^−1^) and 3236 cm^−1^ ($\Delta \bar{v}$ = −23 cm^−1^) in compounds **1**–**3**, respectively. This alteration is typical of the coordination of the metal to the nitrogen atom of the -NH_2_ group. The absorptions in the IR spectra that were related to the vibrations associated with the pyridine ring of the INH did not exhibit significant shifts to higher frequencies after coordination, suggesting that the heterocyclic nitrogen atom does not participate in coordination. The ν(C = O) band in compounds **1**–**3** was displaced to a lower frequency, from 1667 (free ligand) to 1653, 1606 and 1615 cm^−1^, respectively, suggesting coordination by the oxygen atom of the carbonyl group. The presence of hydration water in compounds **1**–**3** is clearly detected by the appearance of its characteristic absorptions at 3500–3600 cm^−1^ (νOH) and 1655 cm^−1^ (δHOH) [14].

The IR spectra of the compound **2** exhibit a very intense band at approximately 2130 cm^−1^, which was attributed to the ν_as_(NCS) vibrational mode, the typical spectral range of the terminal coordination by the sulfur atom [16].

The shift of the 2212, 1317 and 620 cm^−1^ bands of the cyanate group related to the ν_as_(CN), ν_s_(CO) and δ(NCO) vibrational modes, respectively, for compound **3** confirms the coordination of the pseudohalide of terminal mode by the nitrogen atom [17].

#### 2.1.2. Ultraviolet-Visible (UV-Vis) Spectroscopy Studies

The electronic absorption data of the Cu(II) compounds and their attributions are listed in Table 2. The strong bands observed at 249–273 nm were assigned to the Intraligand Transition (IL) of the isoniazid ligand. The Ligand to Metal Charge Transfer (LMCT) transitions of the Cl^−^→Cu(II) [18], NCS^−^→Cu(II) [19] and NCO^−^→Cu(II) [20] types were detected at ~352, 353 and 356 nm, respectively. The appearance of a broad and very low intensity band at 678–688 nm was assigned to the spin-allowed ^2^E_g_←^2^T_2g_ transition, which is typical of octahedral Cu(II) compounds (*d*^9^ electronic systems) [21].

**Table 2:** UV-Vis spectral data ^a^ of INH and the Cu(II) complexes (1.0 × 10^−3^ mol·L^−1^).

Compound	IL Transition	LMCT Transition	LF Transition	Ref.
INH	249 (3120)	-	-	This work
[CuCl_2_(INH)_2_]·H_2_O (**1**)	259 (2930)	352 (1170)	678 (228)	This work
[Cu(NCS)_2_(INH)_2_]·5H_2_O (**2**)	249 (3000)	353 (1000)	688 (184)	This work
[Cu(NCO)_2_(INH)_2_]·4H_2_O (**3**)	273 (1440)	356 (1120)	678 (233)	This work

^a^ absorption maximum in nm and ε in L·mol^−1^·cm^−1^; IL = intraligand; LMCT = Ligand to metal charge transfer; LF = ligand field.

#### 2.1.3. Electronic Paramagnetic Resonance (EPR) Studies and Proposed Structures for the New Complexes

**Figure 5:**
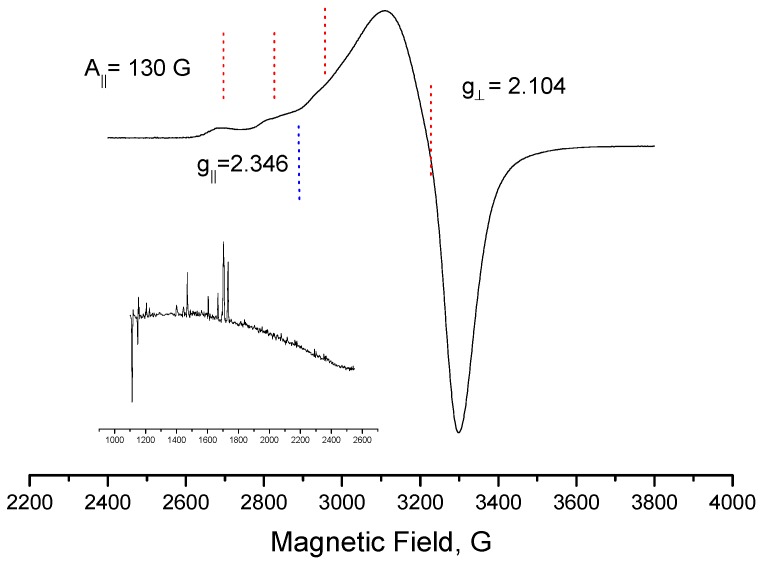
EPR spectra for the [CuCl_2_(INH)_2_]·H_2_O complex (**1**) registered in a frozen DMSO solution at 77 K.

An analysis of the EPR result (Figure 5) of compound **1** allows us to suggest that the Cu(II) ions were octahedrally coordinated, with the octahedron elongated in the axial direction, because neither of the g values is equal to 2.00 [22]. The literature reports that the EPR spectra of divalent copper species obtained in solution at a temperature of 77 K, which have g_║_ > g_⊥_ > g_0_, are typical Cu(II) (*d*^9^) in axial symmetry, with the unpaired electron in the orbital $d_{x^{2}-y^{2}}$[23]

Thus, it is suggested that complex **1** displays an octahedral symmetry around the copper ion with strong tetragonal distortion due to the Jahn Teller effect. Its coordination sites are occupied by two chloro atoms at the axial positions and two molecules of isoniazid in the equatorial plane via the nitrogen atom of the -NH_2_ group and the oxygen atom of the carbonyl group to form five-membered chelate rings (Figure 2).

For complexes **2** and **3**, it is suggested that the compounds consist of monomeric species, in which the environment around the copper ion is octahedral, with some tetragonal distortion. As shown in Figure 3 and Figure 4 (the water molecules are omitted), it is suggested that the coordination sphere of the compounds, with the minimum formula [CuX_2_(INH)_2_] (X = SNC^−^ (**2**) and NCO^−^ (**3**)), consists of two isoniazid ligands coordinated by the nitrogen atom of the -NH_2_ group and the carbonyl oxygen atom, forming two five-membered chelate rings and two pseudohalide groups. For compound **2**, the two thiocyanate groups are likely terminally coordinated by the sulfur atoms, whereas the cyanate pseudohalides are terminally linked by the nitrogen atoms in compound **3**.

### 2.2. Nanostructured Lipid System

Table 3 shows the mean values and standard deviation of the particle size and polydispersity index (PDI) for the nanostructured lipid system (ME) and the copper(II) compounds loaded into the microemulsion. PDI measures the homogeneity between the average particle size in relation to the standard deviation, where smaller PDI values represent more uniform particles [24].

**Table 3:** Determination of the droplet size and polydispersity index of the nanostructured lipid system (MEs) using light scattering.

Formulation	Mean Diameter ± S.D. (nm) *	Mean PDI ± S.D. *
ME	125.6 ± 1.804	0.246 ± 0.008
1-Loaded	158.0 ± 1.060	0.228 ± 0.011
2-Loaded	212.6 ± 1.539	0.284 ± 0.034
3-Loaded	171.7 ± 1.947	0.218 ± 0.007

* Standard deviation (S.D.), polydispersity index (PDI).

The diameter of the ME particles was 125.6 ± 1.804 nm. After the incorporation of the complexes, there was a small variation in the particle diameter size, ranging from 158.0 ± 1.060 to 212.6 ± 1.539 nm. All values are smaller than 1.0 μm, which is characteristic of one ME [25]. When comparing the MEs and the formulations containing the complexes, there was a small increase in the particle diameter, a strong indication that the complexes were incorporated into the nanostructured lipid system. The PDI values of the nanostructured lipid system before and after the incorporation of the coordination compounds varied between 0.218 ± 0.007 and 0.284 ± 0.034, indicating a good size distribution of the droplets in the ME system.

### 2.3. In Vitro Biological Activity

The *in vitro* results (*i.e.*, the MIC and IC_50_ values) obtained for the free copper(II) complexes and those loaded into the microemulsions are shown in Table 4.

**Table 4:** Results of biological assessments (MIC and IC_50_) and determination of the selectivity index (SI) of the free copper(II) complexes or those loaded into the nanostructured lipid system against *S. aureus* and *E. coli.*

Complexes	Parameters					
	*S. aureus*			*E. coli*		
	MIC (μg/mL)	IC_50_ (μg/mL)	SI	MIC (μg/mL)	IC_50_ (μg/mL)	SI
1-Not loaded	1000	109.5	0.1095	2000	109.5	0.05475
1-Loaded	250	319.3	1.288	125	319.3	2.554
2-Not loaded	500	314.3	0.6286	2000	314.3	0.1571
2-Loaded	500	329.3	0.6586	125	329.3	2.634
3-Not loaded	1000	325.3	0.3253	8000	325.3	0.040
3-Loaded	125	>500	4.000	500	>500	1.000

Before loading into the nanostructured lipid system, the compounds had shown moderate activity against *S. aureus*; compounds **1** and **3** showed MICs of 1000 μg/mL, and compound **2** displayed an MIC of 500 μg/mL. Their activity against *E. coli* was even lower as compounds **1**, **2** (MIC 2000 μg/mL) and **3** (8000 μg/mL) showed higher MIC values. After being loaded into the nanosystem, compounds **1** and **3** displayed improved antibacterial activity against *S. aureus* as their MIC values were reduced eight-fold; compound **2** did not display improved activity, even after incorporation. We highlighted the results against *E. coli* as all the compounds displayed improved antibacterial activity, with MIC values that were 16-fold lower than those before incorporation. Compounds **1** and **2** displayed MIC values from 2000 to 125 μg/mL and compound **3** displayed MIC values from 8000 to 500 μg/mL.

The compound **1** and **2**-loaded MEs showed an MIC of 125 μg/mL against *E. coli*, which is better than the [Cu(TTA)_2_], TTA = 2-tenoyltrifluoroacetone compound studied by Xu *et al.* [7] that showed an MIC of 180 μg/mL.

Regarding the activity against *S. aureus*, when compound **3** was loaded into the nanosystem, it exhibited an MIC of 125 μg/mL, with better antibacterial activity than [Cu(L)(ClO_4_)(H_2_O)] (HL = 4-[(*E*)-(2-hydroxy-4-methoxyphenyl)methyleneamino]benzoate), which exhibited an MIC of 500 μg/mL [6]. There are no reports of the use of a nanostrucutured lipid system as a strategy to improve the antibacterial activity of copper(II) complexes against *E. coli* and *S. aureus*.

Moreover, as shown in Table 2, the cytotoxicity results showed that the cytotoxicity of all compounds was after they were loaded into the nanosystem, particularly compound **3** (IC_50_ from 325.3 to higher than 500). The compounds also exhibited higher selectivity index values, even though they still are not ideal (SI higher than 10).

## 3. Experimental Section

### 3.1. General Information

Amphotericin-B (AMB), cholesterol (CHO), CuCl_2_·2H_2_O, isoniazid (INH), polyoxyethylene-20 cetyl ether (Brij^®^ 58), potassium cyanate and rezasurin were purchased from Sigma-Aldrich (St. Louis, MO, USA). Methanol, dimethylsulfoxide (DMSO), dimethylformamide (DMF) and tetrahydrofuran (THF) were purchased from Merck Sharp & Dohme Ltd. (Darmstadt, Hesse, Germany). Dulbecco’s modified Eagle’s medium (DMEM), fetal bovine serum, gentamicin sulfate, and trypsin/EDTA were purchased from Vitrocell Embriolife (Campinas, SP, Brazil). Soy phosphatidylcholine was purchased from Lipoid (Newark, NJ, USA). All the solutions were prepared using deionized water from a Millipore instrument (Darmstadt, Hesse, Germany).

### 3.2. Synthesis of the Copper(II) Complexes

#### 3.2.1. [CuCl_2_(INH)_2_]·H_2_O (**1**)

Approximately 80.4 mg (0.586 mmol) of isoniazid was dissolved in 5 cm^3^ of CH_3_OH and added dropwise to a green solution of CuCl_2_·2H_2_O (200 mg; 0.993 mmol) in 15 cm^3^ of the same solvent, producing a light green suspension. After stirring for 3 h, the solid was filtered, washed with methanol and vacuum-dried at room temperature. Yield: 52%. The compound is soluble in dimethylsulfoxide and dimethylformamide. M.p. = 153 °C (dec.). Anal. Calcd for C_12_N_6_H_14_O_2_Cl_2_Cu (%): C, 33.8; N, 19.7; H, 2.38; Cu, 15.6. Observed: C, 31.8; N, 21.4; H, 2.47; Cu, 15.3 [26].

#### 3.2.2. [Cu(NCS)_2_(INH)_2_]·5H_2_O (**2**)

Approximately 161 mg (1.17 mmol) of isoniazid was dissolved in 10 cm^3^ of CH_3_OH and added to a solution of 50 mg (0.293 mmol) of CuCl_2_·2H_2_O in 15 cm^3^ of the same solvent. After stirring this suspension for 5 min, 47.5 mg (0.0586 mmol) of sodium thiocyanate (NaSCN), dissolved in 5 cm^3^ of water was added to the mixture and stirred for 3 h. The dark green solid was isolated by filtration, washed with methanol and vacuum-dried at room temperature. The compound is soluble in methanol, dimethylsulfoxide, dimethylformamide and tetrahydrofuran. Yield: 40%. M.p. = 149 °C. Anal. Calcd for C_14_N_8_H_24_O_7_S_2_Cu (%): C, 30.9; N, 20.6; H, 4.45; Cu, 11.7. Observed: C, 31.5; N, 20.2; H, 2.46; Cu, 11.0.

#### 3.2.3. [Cu(NCO)_2_(INH)_2_]·4H_2_O (**3**)

A solution of 161 mg (1.17 mmol) of isoniazid in 10 cm^3^ of CH_3_OH was added to a solution of 50 mg (0.293 mmol) of CuCl_2_ 2H_2_O in 15 cm^3^ of the same solvent. After stirring this suspension for 5 min, 47.6 mg (0.0587 mmol) of potassium cyanate (KNCO) dissolved in 5 cm^3^ of water was added to reaction medium, producing a dark blue suspension. The mixture was stirred for 5 h, and the resulting precipitate was isolated by filtration. The product was thoroughly washed with methanol and dried in vacuum at room temperature. The compound is soluble in methanol, dimethylsulfoxide, dimethylformamide and tetrahydrofuran. Yield: 33%. M.p. ≥ 250 °C. Anal. Calcd. for C_14_N_8_H_22_O_8_Cu (%): C, 34.0; N, 22.7; H, 4.49; Cu, 13.0. Observed: C, 34.0; N, 18.0; H, 3.06; Cu, 13.6.

### 3.3. Characterization of the Copper(II) Complexes

#### 3.3.1. IR Spectroscopy

The vibrational spectra in the infrared region were registered on a Nicolet FTIR-Impact 400 spectrometer (Thermo Scientific, San Jose, CA, USA), in the range 400–4000 cm^−1^. The samples were prepared as KBr pellets (~10 mg complex/100 mg KBr) that had previously been dried at 120 °C.

#### 3.3.2. UV-Vis Spectroscopy

The electronic spectra were recorded on a Lambda 14P spectrophotometer (Perkin Elmer, Boston, MA, USA) using quartz cells with a 1.00 cm optical length and 1.0 × 10^−3^ mol·L^−1^ solutions of the complexes in methanol.

#### 3.3.3. EPR Spectroscopy

The EPR spectra were recorded on a Bruker EMX instrument (Bruker, Karlsruhe, Germany) working at X-band (9.65 GHz frequency, 20 mW power, 100 kHz modulation frequency). DPPH (α,α′-diphenyl-β-picrylhydrazyl) was used as a magnetic field calibrator (g = 2.0036), and the measurements were performed with a frozen dimethylsulfoxide solution of complex **1** in Wilmad quartz tubes (4 mm internal diameter). Usually, the standard conditions for recording the spectra were 15 G modulation amplitude, 7.96 × 10^3^ or 3.56 × 10^4^ receiver gain, and 2 or 4 scans at 77 K.

#### 3.3.4. Melting Point Determination

The melting points of the samples (~5 mg complex) were determined using the MQAPF-302 instrument (Microcriquímica Equipamentos Ltda, Palhoça, Santa Catarina, Brazil), which reaches a maximum temperature of 350 °C.

#### 3.3.5. Elemental Analysis

The elemental analyses were performed at the Central Analitica at IQ University of São Paulo, Brasil using a CNH 2400 Perkin-Elmer instrument (Perkin Elmer), which determines the percentage of carbon, hydrogen and nitrogen in the samples with a precision of ±0.5%.

#### 3.3.6. Complexometry with 2,2′,2′′,2′′′-(Ethane-1,2-diyldinitrilo)tetraacetic Acid (EDTA)

To analyze the copper content, 5.00 mg of the samples were weighed on an analytical balance with an uncertainty of 0.01 mg. The sample was digested by the addition of five drops of a hot HNO_3_ solution (70% *w*/*w*). After cooling, 1.0 mL ammonium acetate buffer solution (0.2 mol·L^−1^) and a known excess quantity of EDTA solution that was sufficient to complex all the metal present in the sample were added. The pH was adjusted to 5.0 (±0.1) and the xylenol orange indicator was added in a sufficient quantity to observe a yellow color. Then, the solution was subjected to titration with a ZnCl_2_ solution (0.01 mol·L^−1^), which was previously standardized with EDTA. The turning point was observed by the appearance of pink color. The copper content was calculated using the following equation:

C_Cu_ = M_Cu_ × (C_EDTA_ × V_EDTA_ − C_ZnCl2_ × V_ZnCl2_)/m_sample_

where C_Cu_ = the copper content; M_Cu_ = the molar mass of copper; C_EDTA_ = the concentration of the EDTA solution; V_EDTA_ = the volume of the EDTA solution; C_ZnCl2_ = the concentration of the ZnCl_2_ solution; V_ZnCl2_ = the volume of the ZnCl_2_ solution; and m_sample_ = the mass of the weighed sample [27].

### 3.4. Nanostructured Lipid System Preparation

The MEs were prepared as described by Bonifácio *et al.* [28], with the following composition: 10% oil phase (*i.e.*, cholesterol), 10% surfactant (*i.e.*, polyoxyethylene-20 cetyl ether (Brij^®^ 58) and soy phosphatidylcholine in a proportion of 2:1) and 80% aqueous phase (*i.e.*, phosphate buffer, pH 7.4). The mixture was sonicated using a rod sonicator (Q700 of QSonica^®^, Newtown, CT, USA) at 700 watts in discontinuous mode for 10 min with 30 s incubations in an ice bath every two minutes during the sonication process. After sonication, the MEs were centrifuged at 11,180× *g* for 15 min to eliminate the waste released by the titanium rod sonicator.

### 3.5. Nanostructured Lipid System Characterization: Mean Diameter and Polydispersity Index (PDI)

The microemulsion droplet size and distribution were determined by an optical particle analyzer (Zetasizer Nano-ZS ZEN3600, Malvern Instruments, San Diego, CA, USA) using dynamic light scattering. All samples (MEs with and without inorganic compounds) were diluted in Milli-Q water (100 μL of sample in 900 μL of deionized water) and placed in the instrument. The analyses were performed in triplicate.

### 3.6. Preparation of the Coordination Compound-Loaded Nanostructured Lipid System

After obtaining the MEs, the copper(II) complexes were loaded into the nanostructured lipid system. A complex (0.0100 g) was added to the MEs (2 mL) and the mixture was homogenized and sonicated for 5 min at room temperature in discontinuous mode to facilitate the incorporation of the material into the microemulsion at a concentration of 5000 μg/mL. The inorganic compound-loaded nanostructured lipid systems were characterized by measuring the mean diameter and polydispersity index.

### 3.7. Antibacterial Activity

The antibacterial activity against *Staphylococcus aureus* (ATCC 25923) and *Escherichia coli* (ATCC 25922) was determined by the microdilution method, according to the standard reference method M7-A6 CLSI (2006) [29]. Both of the strains used in this study were obtained from the American Type Culture Collection (Rockville, MD, USA). Compounds **1**, **2** and **3** were loaded into the microemulsions at an initial concentration of 2000 μg/mL. At first, 100 μL of each compound loaded into the nanosystem were added to 96-well microplates containing 80 μL/well of Mueller-Hinton broth, and then a two-fold serial dilution was performed to obtain concentrations ranging from 7.81 μg/mL to 8000 μg/mL. Ampicillin and the microemulsions without the compound were used as positive and negative controls, respectively. The bacterial inocula were standardized at 1.0 × 10^7^ CFU/mL and the microplates were incubated at 37 °C for 24 h. Then, 30 μL/well of a resazurin solution (0.01%) were added, and, after incubation at 37 °C for 2 h, the minimum inhibitory concentration (MIC) was determined. Bacterial growth changes the blue resazurin dye to pink, and the blue color indicates growth inhibition. MIC was defined as the lowest concentration that is able to inhibit at least 90% of bacterial growth. All tests were performed in triplicate.

### 3.8. In Vitro Cytotoxic Activity

The cytotoxicity of the free complexes diluted in DMSO and those incorporated into the nanostructured lipid systems was measured on normal epithelial cells (VERO ATCC CCL-81) as described by Pavan *et al.* [30]. The cells were seeded on plates with a surface area of 12.50 cm^2^ in 10 mL DMEM supplemented with 10% fetal bovine serum, gentamicin sulfate (50 mg/L) and amphotericin B (2 mg/L) and incubated at 37 °C and 5% CO_2_.

This technique consists of collecting the cells using a solution of trypsin/EDTA, centrifugation (2000 rpm for 5 min), counting the number of cells in a Newbauer chamber and then adjusting the concentration to 3.4 × 10^5^ cells/mL using DMEM [31]. Next, 200 μL of the suspension was deposited into each well of a 96-well microplate to obtain a cell density of 6.8 × 10^4^ cells/well and incubated at 37 °C in an atmosphere of 5% CO_2_ for 24 h to allow the cells to attach to the plate. Dilutions of the test compounds were prepared to obtain concentrations from 500 to 1.95 μg/mL. The dilutions were added to the cells after the media was removed, and any cells that did not adhere were incubated for an additional 24 h. The cytotoxicity of the compounds was determined by adding 30 μL of resazurin developer and read after a six-hour incubation. The samples were analyzed using a microplate Spectrafluor Plus (TECAN^®^) reader and excitation and emission wavelengths of 530 and 590 nm, respectively. The cytotoxicity (IC_50_) was defined as the highest concentration of the compound that retained the viability of at least 50% of the cells.

### 3.9. Selectivity Index (SI)

The selectivity index (SI) can be calculated by dividing the IC_50_ value by the MIC value. An SI greater than or equal to 10 indicates that the test compound can be applied at a concentration that is ten-fold higher than the MIC value without exhibiting cytotoxicity [32].

## 4. Conclusions

The synthesis, characterization, and antibacterial activity of copper(II) complexes bearing an isoniazid ligand that were incorporated into nanostructured lipid system were reported in this work. The nanostructured lipid system was used as a strategy to improve the antibacterial activity against *E. coli* and *S. aureus* and decrease the cytotoxicity of the studied compounds. Our findings suggested that the Cu(II) complexes may be promising antibacterial candidates when they are incorporated into the MEs described in this study.
